# Exploring the Anti‐Nociceptive, Anti‐Inflammatory, Anti‐Pyretic, and Anti‐Arthritic Activity of Methanolic Extract of *Desmos chinensis* and Its Different Solvent Fractions: In Vivo, In Vitro, and In Silico Intervention

**DOI:** 10.1155/adpp/3277644

**Published:** 2026-05-14

**Authors:** Nigar Sultana Prithy, Ainun Nahar, Md. Jahirul Islam Mamun, Md. Hossain Rasel, Fahmida Akter, Kazi Sanjida Tahrim, Raina Islam, Md. Emdadul Hossain Emon, Abu Bin Ihsan

**Affiliations:** ^1^ Department of Pharmacy, Faculty of Biological Sciences, University of Chittagong, Chittagong, 4331, Bangladesh, cu.ac.bd; ^2^ Department of Pharmacy, School of Life Sciences, Eastern University, Dhaka, 1345, Bangladesh, esn.ac.lk; ^3^ Department of Pharmacy, International Islamic University Chittagong, Chittagong, 4318, Bangladesh, iiuc.ac.bd; ^4^ Department of Biochemistry and Molecular Biology, University of Dhaka, Dhaka, 1000, Bangladesh, du.ac.bd

**Keywords:** analgesic, antiarthritic, anti-inflammatory, anti-pyretic, *Desmos chinensis*

## Abstract

**Objective:**

*Desmos chinensis* (Annonaceae) is an underexplored medicinal plant from Southeast Asia with limited pharmacological validation. This study investigated, for the first time, the analgesic, anti‐pyretic, anti‐inflammatory, and anti‐arthritic potential of the methanolic extract of *D. chinensis* leaf and its solvent‐soluble fractions (n‐hexane, dichloromethane, and ethyl acetate) using in vivo, in vitro, and in silico approaches.

**Methods:**

Analgesic activity was assessed using the acetic acid–induced writhing method; anti‐pyretic activity was assessed using the brewer’s yeast‐induced pyrexia model; anti‐inflammatory activity was assessed using protein denaturation; and anti‐arthritic activity was assessed using the human red blood cell membrane stabilization method.

**Result:**

In the writhing test, the ME and ethyl acetate fractions at 400 mg/kg produced marked analgesic effects, with 86.36% and 65.91% inhibition of writhing, respectively, compared with diclofenac (90.45%). In the anti‐pyretic model, ME significantly reduced yeast‐induced hyperthermia, achieving up to 100% reduction in pyrexia within 4 h at 400 mg/kg (*p* < 0.001). In vitro assays showed concentration‐dependent anti‐inflammatory and anti‐arthritic activities. ME inhibited protein denaturation with an IC_50_ of 337.42 μg/mL, while HRBC membrane stabilization revealed strong anti‐arthritic potential with an IC_50_ of 246.34 μg/mL, comparable to diclofenac sodium. Docking analysis revealed favorable interactions between phytoconstituents and inflammation‐related targets.

**Conclusion:**

This study provides the first integrated pharmacological evidence that *D. chinensis* leaves possess significant analgesic, anti‐pyretic, anti‐inflammatory, and anti‐arthritic activities. The quantitative efficacy and in silico findings highlight *D. chinensis* as a promising source of bioactive compounds, warranting further isolation, mechanistic, and preclinical investigations.

## 1. Introduction

Pain is an unpleasant experience that involves both sensory and emotional aspects, linked to actual or potential harm to body tissues, or described in relation to such harm [1]. It is typically triggered by harmful stimuli and carried through specialized nerve pathways to the central nervous system, where it is perceived. It serves as a protective mechanism to prevent potential injury to the body [2]. Likewise, inflammation is a defensive response used by both the innate and adaptive immune systems, activated in reaction to harmful stimuli such as infections or tissue damage [3, 4]. These conditions are marked by classic clinical signs such as redness, swelling, and warmth, which arise due to the release of various mediators, including kinins, platelet‐activating factor, prostaglandins, leukotrienes, amines, cytokines, and chemokines [5]. Currently, pain management through pharmacotherapy depends on a range of drugs, including non‐steroidal anti‐inflammatory drugs (NSAIDs) and opioid analgesics [6]. However, these medications can cause numerous side effects. For instance, NSAIDs may lead to gastric ulcers, stomach irritation, changes in blood pressure, impaired kidney and liver function, and platelet inhibition [7]. Conversely, opioids are frequently associated with adverse effects such as dependence, sedation, constipation, and respiratory complications. Meanwhile, medicinal plants have long been a valuable source of bioactive compounds, many of which offer considerable potential for drug discovery and continue to act as lead compounds in pharmaceutical development [8]. Therefore, investigating medicinal plants recognized for their analgesic and anti‐inflammatory properties may provide opportunities to identify new compounds with enhanced safety and efficacy.

Pyrexia, or fever, commonly arises from infections, tissue injury, inflammation, graft rejection, malignancies, or other pathological conditions. As a natural defense mechanism, the body raises its temperature to create conditions unfavorable for infectious agents or damaged tissue. Typically, infected or injured tissues stimulate the production of pro‐inflammatory mediators, such as cytokines (interleukin‐1β, IL‐1α, IL‐1β, and TNF‐α), which in turn increase prostaglandin E2 (PGE2) synthesis near the preoptic area of the hypothalamus, prompting the hypothalamus to raise body temperature [9, 10]. Most anti‐pyretic drugs lower elevated body temperature by preventing or inhibiting COX‐2 expression, thereby reducing PGE2 production. Synthetic agents, however, often irreversibly inhibit COX‐2 with high selectivity, which can lead to toxicity in the liver, kidney glomeruli, brain cortex, and heart. In contrast, natural COX‐2 inhibitors generally exhibit lower selectivity and are associated with fewer adverse effects [11].

Arthritis, an autoimmune disorder, is characterized by pain, stiffness, and swelling. It results from inflammation of the synovial joints due to an immune‐mediated response. Globally, approximately one in five older adults is affected by arthritis [12]. A variety of drugs have been employed to manage arthritis, including NSAIDs, sulfasalazine, D‐penicillamine, cyclophosphamide, cyclosporine, methotrexate, azathioprine, glucocorticoids, as well as biologics such as anakinra, etanercept, abatacept, and infliximab [13]. However, these treatments carry various risks, including gastrointestinal ulcers, stomatitis, cardiovascular complications, pulmonary toxicity, myelosuppression, hematologic and kidney toxicity, liver fibrosis, cirrhosis, diarrhea, and localized reactions at injection sites [14]. Therefore, developing new anti‐arthritis drugs derived from affordable medicinal plants with minimal side effects is highly important.

For centuries, plants have served as an essential source of natural products for promoting human health, and over the past decade, research on plant‐based therapies has intensified [15]. The World Health Organization recognizes medicinal plants as a prime source for developing a wide range of drugs [16]. Approximately 80% of people in developing countries rely on traditional medicine, which often contains compounds derived from medicinal plants. Consequently, these plants should be thoroughly studied to evaluate their properties, safety, and efficacy [17].

*Desmos chinensis* Lour. (family: Annonaceae), a flowering shrub native to Southeast Asia, including Thailand, Malaysia, and regions of India, has recently attracted pharmacological interest. Traditionally, its bark and leaves have been used to manage fever, wounds, infections, and gastrointestinal disorders [18]. Initial phytochemical analyses have revealed the presence of compounds including flavonoids (such as mateucinol, negletein, and lawina), chalcones, essential oils, and alkaloids, many of which are linked to anti‐inflammatory, antioxidant, and cytoprotective activities [19, 20]. Despite these promising constituents, systematic pharmacological validation of the leaves remains limited and fragmented, and most reports focus mainly on isolated chemical profiling, antioxidant and antimicrobial activity, rather than integrated biological efficacy [18, 21].

Importantly, no previous study has comprehensively evaluated the analgesic, anti‐pyretic, anti‐inflammatory, and anti‐arthritic activities (traditionally claimed activities) of the methanolic leaf extract of *D. chinensis* using combined in vivo, in vitro, and in silico models. Furthermore, its potential as a functional botanical ingredient for the treatment of inflammation‐related conditions has not yet been explored.

This study aims to fill a crucial research gap by systematically assessing the methanolic leaf extract of *D. chinensis* for its multiple pharmacological effects through a combination of validated in vivo, in vitro, and in silico approaches. To the best of our knowledge, this is the first comprehensive pharmacological evaluation of *D. chinensis*, linking its traditional medicinal uses with scientific evidence and laying the groundwork for its potential development as a plant‐based therapeutic or nutraceutical for the management of inflammation‐related conditions.

## 2. Materials and Methods

### 2.1. Chemicals and Reagents

For analgesic and anti‐inflammatory tests, diclofenac sodium (Square Pharmaceuticals Ltd., Dhaka, Bangladesh) served as the reference. Merck (Darmstadt, Germany) supplied analytical‐grade fractionation solvents, including n‐hexane, ethyl acetate, acetic acid, and methanol. The remaining chemicals were all of analytical grade and did not require any additional purification.

### 2.2. Plant Materials Collection and Identification

Freshly picked leaves of *D. chinensis* were collected from Chattogram, Bangladesh. The specimen was identified as *D. chinensis* by Dr. Shaikh Bokhtear Uddin, taxonomist and professor in the Department of Botany.

### 2.3. Extraction and Partitioning Process

The plant materials were sun‐dried in a semi‐shed for 7 days and then finely ground using a high‐performance electric grinder (Brand: Damai). They were subsequently soaked in 2.5 L of methanol for 14 days at room temperature (23 ± 0.5°C). The mixture was filtered through cheesecloth and Whatman No. 1 filter paper, and the filtrate was concentrated under reduced pressure at temperatures below 45°C using a rotary evaporator (RE200, Bibby Sterilin, Stone, Staffordshire, UK). The solvent was removed entirely, and the resulting crude extract was stored in airtight containers for further analysis. The crude extracts were subsequently subjected to solvent–solvent partitioning following the modified method of Kupchan [22] using three solvents: n‐hexane, ethyl acetate, and dichloromethane, using their ascending polar characteristics, including n‐hexane/NH (non‐polar), dichloromethane/DCM (moderately polar), and ethyl acetate/EA (polar).

### 2.4. Qualitative Screening

Preliminary phytochemical screening was performed to detect the presence of terpenoids, flavonoids, saponins, phenols, tannins, phlobatannins, steroids, anthraquinones, alkaloids, glycosides, cardiac glycosides, resins, carbohydrates, proteins, fats, oils, and coumarins using standard methods [23].

### 2.5. Experimental Animal

The study employed 4–5‐week‐old male Swiss albino mice (*Mus musculus*) weighing 20–25 g, obtained from the animal House at Jahangirnagar University, Dhaka. The mice were housed in clean, dry polypropylene cages under controlled conditions, with a 12‐h light/dark cycle, temperature of 25 ± 2°C, and relative humidity of 45%–55%. The animals were acclimated to the laboratory environment for 3‐4 days before experimentation, fed a standard laboratory diet, and provided water ad libitum. To standardize experimental conditions, food was withheld for 12 h before and during the procedures. All experimental protocols adhered to ethical guidelines for the care and use of laboratory animals.Ethical approval was obtained from theInstitutional Ethics Review Board (IERB) of Eastern University, Dhaka,Bangladesh, under approval no. IERB‐2024‐0005. At the end of the experimentalprocedures, animals were humanely euthanized using an overdose of ketamine (150mg/kg) and xylazine (15 mg/kg) administered intraperitoneally. The euthanasiaprocedure was carried out in accordance with the AVMA Guidelines for the Euthanasia of Animals to ensure minimalpain and distress to the animals.

### 2.6. Experimental Design

Sample sizes were determined based on previously published studies in analgesic and anti‐pyretic models and were calculated to provide adequate statistical power (*n* = 5 per group) to detect significant differences with a confidence level of 95% (*p* < 0.05). For in vivo analgesic assessment, 40 mice were randomly selected and divided into 8 groups of 5 animals each. Group I served as the control, receiving 1% Tween‐80 and DMSO in saline (10 mL/kg, p.o.), while Group II received the standard drug, diclofenac sodium (50 mg/kg, p.o.). Groups III–VIII were treated orally with either 200 or 400 mg/kg of the methanolic extract (ME) or its n‐hexane, dichloromethane, and ethyl acetate fractions, respectively. For the in vivo anti‐pyretic evaluation, 20 mice were randomly assigned to 4 groups of 5 animals each. Group I received the control (1% Tween‐80 and DMSO in saline, 10 mL/kg, p.o.), Group II received the standard drug paracetamol (150 mg/kg), and Groups III and IV were treated with the methanolic leaf extract at doses of 200 mg/kg and 400 mg/kg, respectively. All treatments and measurements were performed by investigators blinded to the group assignments to reduce experimental bias. The experimental endpoints (writhing responses and rectal temperature) were recorded by personnel unaware of the treatments. Ethical approval was obtained from the Institutional Ethics Review Board (IERB) of Eastern University, Dhaka, Bangladesh, under approval no. IERB‐2024‐0005.

### 2.7. Acute Toxicity Evaluation and Dose Determination

To verify the extract’s safety limit before the trial, we carried out a pilot acute toxicity evaluation using the OECD 425 guideline (Limit test) as described in the work by Khandakar et al. [24]. Five fasted female mice (non‐pregnant) weighing 25–30 g were administered an oral dose of ME at a dose of 2 g/kg body weight. After administration, the animals were fasted for an additional 3‐4 h. Following oral treatment, the mice were monitored for changes in eyes, mucous membranes, skin, fur, respiration, circulation, and autonomic and central nervous system functions. Observations were conducted immediately during the first 30 min, then at 24‐h intervals, with particular attention to the first 4 h, and continued for a total of 14 days to detect any delayed toxicity. Each animal’s body weight was measured before treatment (Day 0) and at regular intervals (Days 7 and 14) during the observation period to monitor any changes. The therapeutic dose was set at one‐tenth of the median lethal dose (LD_50_). The percentage of body‐weight change was calculated using the following formula:

(1) % change=Day 140 weight−Day  weightDay 0 weight×100.

Sudden weight loss of each mouse (> 10%) indicates toxicity of the extract.

### 2.8. Analgesic Activity

#### 2.8.1. Acetic Acid–Induced Writhing Method

The acetic acid–induced writhing test was conducted as previously documented by Mamun‐Or‐Rashid et al. [25]. Each group received a specific treatment as described in the experimental design section. After 30 min of receiving this treatment, 0.7% acetic acid was injected intraperitoneally at a dosage of 10 mL/kg body weight. The number of abdominal contractions (writhes) was recorded for each group of mice from 5 min post–acetic acid injection to 20 min, and the results were reported as a percentage of protection. The percentage protection against acetic acid was calculated using the following formula:

(2) % of inhibition=Nc−NtNc×100,

where Nc = number of writhings in control and Nt = number of writhings in test animals.

### 2.9. Antipyretic Activity

#### 2.9.1. Brewer’s Yeast‐Induced Pyrexia Method

For this study, the methodology mentioned by Sahu et al. [26] was applied. Rectal temperature was measured at predetermined intervals to assess each Swiss albino male mouse’s body temperature. Before the experiments, Swiss albino male mice (*Mus musculus*) were starved overnight and given water ad libitum. The animal’s dorsum region was subcutaneously injected with a 20% (W/V) brewer’s yeast suspension (10 mL/kg) to induce pyrexia. The rectal temperature of each rodent was remeasured 24 h after yeast administration. Mice that failed to demonstrate a minimal temperature increase of 0.5°C 24 h following yeast injection were discarded. The 20 selected mice were immediately treated as described in the experimental design section. The digital thermometer (SK‐1250 MC, Sato Kiriaki Mfg. Co., Ltd., Japan) was inserted into the rectum of each mouse after 30 min. Subsequently, the rectal temperature was recorded. The percentage of reduction in pyrexia was then calculated using the following formula:

(3) % of reduction of pyrexia=B−CB−A×100,

where *A* = normal body temperature; *B* = rectal temperature at 24 h after yeast administration; and *C* = rectal temperature after drug administration at a different time interval.

### 2.10. Anti‐Inflammatory Activity

#### 2.10.1. Egg Albumin Denaturation Assay

The assay was carried out as described by Sakat et al. [27] with modifications. The 5‐mL reaction mixture consisted of 0.2 mL of egg albumin (from a fresh hen’s egg), 2.8 mL of phosphate‐buffered saline (pH 6.4), and 2 mL of plant extracts at various concentrations (15.625, 31.25, 62.5, 125, 250, and 500 μg/mL). Double‐distilled water of equal volume served as the control. The mixtures were incubated at 37 ± 2°C for 15 min and then heated to 70°C for 5 min. After cooling, absorbance was measured at 660 nm with the vehicle as the blank. Diclofenac (1 mg/mL) was used as the reference drug and treated similarly for absorbance measurement. The percentage inhibition of protein denaturation was calculated using the following formula:

(4) % inhibition=AC−ASAC×100,

where *A*_*C*_ is the absorbance of the control group and *A*_*S*_ is the absorbance of the sample.

### 2.11. Antiarthritic Activity

The method used was the procedure adopted by Deshpande et al. [28] with slight modifications. Approximately 2 mL of blood was collected from healthy human volunteers (n=2) who had not taken NSAIDs for at least 2 weeks. The 4.5‐mL reaction mixture consisted of 2 mL of hypotonic saline (0.25% NaCl), 1 mL of 0.15 M phosphate buffer (pH 7.4), 1 mL of test solution (0, 100, 200, 400, and 800 μg/mL in normal saline), and 0.5 mL of 10% human red blood cells (HRBC) suspension in normal saline. The mixtures were incubated at 56°C for 30 min and then cooled under running tap water. Following this, the mixtures were centrifuged at 3000 rpm for 10 min, and the absorbance of the supernatant was measured at 560 nm. Methotrexate served as the standard drug. Experiments were performed in triplicate, and the percentage of membrane stabilization activity was calculated using the formula provided as follows:

(5) % inhibition=AC−ASAC×100,

where *A*_*C*_ is the absorbance of the control group and *A*_*S*_ is the absorbance of the sample.

### 2.12. In Silico Study

#### 2.12.1. ADME and Drug‐Likeliness Prediction

The absorption, distribution, metabolism, and excretion (ADME) properties of the bioactive compounds from *D. chinensis* were assessed using Lipinski’s Rule of Five, along with computational tools SwissADME and pkCSM, to evaluate their drug‐likeness and pharmacokinetic profiles [29, 30].

#### 2.12.2. Toxicity Evaluation

The online tools ProTox II (https://tox-new.charite.de/) [31] and admetSAR (https://lmmd.ecust.edu.cn/admetsar1/) [30] were used to assess the toxicity of the phytocompounds. Key toxicological parameters, including immunotoxicity, toxicity class, LD_50_ (median lethal dosage), hepatotoxicity, cytotoxicity, carcinogenicity, and mutagenicity, were predicted using these techniques. The analyses supported the evaluation of the compounds for further pharmacological development by providing insights into their potential safety profiles.

#### 2.12.3. Ligand Preparation

Eighteen minor metabolites of *D. chinensis* [19, 32–34] were selected as ligands for docking. For molecular docking studies, the three‐dimensional (3D) SDF structures of these compounds were retrieved from the PubChem database. In cases where 3D SDF files were not available, two‐dimensional (2D) SDF files were obtained and converted to 3D format using Open Babel software [35]. Before docking simulations, all ligand structures were energy‐minimized and converted into the .pdbqt format using AutoDock Tools (version 1.5.6) to ensure proper preparation for computational docking [36].

#### 2.12.4. Protein Preparation

The RCSB Protein Data Bank provided the crystal structures of the target proteins, which are listed in Table 1. The enzymes’ active sites were determined using data previously published by Kurumbail et al. [37]. Swiss‐PdbViewer (v4.1) and the BIOVIA Discovery Studio 4.5 Client were used to structurally clean the proteins, excluding water molecules, cofactors, and heteroatoms for proper processing [38, 39]. After adding hydrogen atoms, the protein structures were energy‐minimized using the MMFF94 force field in the PyRx virtual screening tool. To ensure compatibility and expedite molecular docking, the generated protein structures were kept in PDB format throughout the docking procedure [40, 41].

**TABLE 1:** Selected protein structures for investigating the pharmacological activities of phytochemicals from *D. chinensis*.

Pharmacological activity	Protein name	PDB ID
Anti‐nociceptive	Human cyclooxygenase‐2	5IKR
Anti‐inflammatory	Human cyclooxygenase (hCOX)‐1	6Y3C
Anti‐pyretic	Microsomal prostaglandin E synthesis 1 (mPGES‐1)	4YK5
Anti‐arthritic	TNF‐alpha with a small‐molecule inhibitor	2AZ5

#### 2.12.5. Molecular Docking Study

PyRx AutoDock Vina 1.2.0, which features new docking algorithms, an expanded force field, and Python bindings, was utilized to dock a selection of proteins with *D. chinensis* ligands. The protein was treated as rigid, and the ligands were treated as semi‐flexible. Root‐mean‐square deviation values < 2 Å were deemed acceptable, and active sites were determined using the co‐crystallized ligand, which was re‐docked to validate the technique. A grid box with a spacing of 0.329 Å and dimensions of *X*: 21.332 Å, *Y*: 34.0472 Å, and *Z*: 18.1073 Å was generated using AutoGrid to precisely define the docking search space centered on the active site of the target protein, ensuring accurate and efficient sampling of ligand binding conformations during molecular docking simulations. BIOVIA Discovery Studio Visualizer 2020 was used to view and evaluate docking interactions [42, 43].

### 2.13. PASS Prediction

The biological activities of *D. chinensis* compounds were predicted using the PASS (Prediction of Activity Spectra for Substances) online server, which estimates the probability of activity (Pa) and likelihood of inactivity (Pi) for various pharmacological effects. The compounds, initially sourced from PubChem, were converted into SMILES format for computational analysis [44, 45]. To evaluate potential therapeutic effects, the search terms “antinociceptive,” “anti‐inflammatory,” “anti‐pyretic,” and “Jak2 expression inhibitor” were used to predict Pa and Pi values corresponding to anti‐nociceptive, anti‐inflammatory, anti‐pyretic, and anti‐arthritic activities, respectively.

### 2.14. Statistical Analysis

All the data were expressed as mean ± SEM (standard error of mean). The results were analyzed statistically using one‐way ANOVA followed by post hoc Dunnett’s *t*‐test, as performed with the Statistical Package for the Social Sciences (SPSS, Version 16.0, IBM Corporation, NY). Results ^∗^ *p* < 0.05, ^∗∗^ *p* < 0.01, and ^∗∗∗^ *p* < 0.001 were considered statistically significant as compared to the control.

## 3. Results

### 3.1. Qualitative Phytochemical Screening

The results of the phytochemical study on the ME of *D. chinensis* are presented in Table 2.

**TABLE 2:** Phytochemical screening results of the methanolic extract of *D. chinensis*.

Tests for phytoconstituents	Test names	Result of ME of *Desmos chinensis*
1. Alkaloids	(a) Dragendroff’s test	+
	(b) Hager’s test	+
	(c) Mayer’s test	+
	(d) Wagner’s test	+

2. Glycosides	(a) Aqueous NaOH test	−
	(b) Conc. sulfuric acid test	+

3. Cardiac glycosides	(a) Keller–Kiliani test	+

4. Protein	(a) Xanthoproteic test	−

5. Reducing sugar	(a) Fehling’s test	+

6. Flavonoids	(a) Alkaline reagent test	−
	(b) Ferric chloride test	+
	(c) Conc. sulfuric acid test	−

*Note:* ‘+’ means present and ‘−’ means absent.

### 3.2. In Vivo Study

#### 3.2.1. Acute Toxicity Evaluation and Dose Determination

For each of the groups, there were no deaths or abnormalities in the test animals at the experimental dosages, including restlessness, convulsions, decreased motor activity, diarrhea, coma, and lacrimation. Therefore, the LD_50_ was found to exceed 2 g/kg body weight. Significant body‐weight changes were observed in the test group at the administered dose. The body changes throughout the observation period are presented in Table 3. To evaluate dose‐dependence, 200 mg/kg was selected as a moderately effective dose and 400 mg/kg as a higher dose, in accordance with other in vivo studies employing plant extracts and safety limits obtained from acute toxicity considerations.

**TABLE 3:** Observation of weight changes in the acute toxicity study.

Animal no.	Day 0 (g)	Day 7 (g)	Day 14 (g)	% Change (day 0–14)
M1	26	26.4	27.3	5.00%
M2	25.4	26.6	27.4	7.87%
M3	26.6	26.9	27.7	4.14%
M4	25.8	26.3	27.2	5.43%
M5	27	27.9	29.7	10.00%

#### 3.2.2. Analgesic Activity by the Acetic Acid–Induced Writhing Method

**FIGURE 1:**
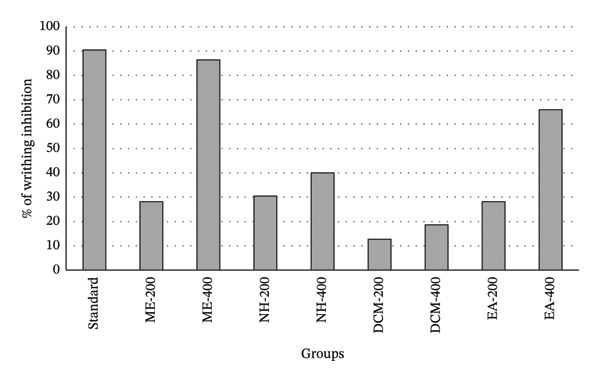
Analgesic activity of methanolic extract and different solvent fractions of *D. chinensis*.

The analgesic potential of the ME and its solvent‐soluble fractions of *Desmos chinensis* leaves was evaluated using the acetic acid–induced writhing model in mice (Table 4 and Figure 1). All the fractions, including the ME, demonstrated a dose–response relationship. At higher doses, the fractions showed a more pronounced effect. The ME of *Desmos chinensis* at 400 mg/kg significantly reduced writhing to 6 ± 1.30 (86.36% inhibition; *p* < 0.001), closely matching the standard diclofenac sodium (4.20 ± 0.86; 90.45% inhibition). The ethyl acetate fraction at 400 mg/kg also exhibited vigorous analgesic activity (15 ± 2.17; 65.9% inhibition; *p* < 0.001).

**TABLE 4:** Analgesic activity of methanolic extract and different solvent fractions of *D. chinensis.*

Group and dose (mg/kg)	Number of writhing (mean ± SEM)
Control	44 ± 2.24
Diclofenac‐10	4.20 ± 0.86^∗∗∗^
ME‐200	31.6 ± 2.14^∗∗∗^
ME‐400	6 ± 1.30^∗∗∗^
NH‐200	30.6 ± 0.87^∗∗∗^
NH‐400	26.4 ± 2.09^∗∗∗^
DCM‐200	38.4 ± 0.97
DCM‐400	35.8 ± 1.01^∗∗^
EA‐200	31.6 ± 1.63^∗∗∗^
EA‐400	15 ± 2.17^∗∗∗^

*Note:* Each value represents the mean ± SEM (*n* = 5). One‐way ANOVA followed by Dunnett’s *t*‐test.

Abbreviations: DCM, dichloromethane fraction; EA, ethyl acetate fraction; ME, methanolic extract; NH, n‐hexane fraction.

^∗∗∗^ *p* < 0.001 and ^∗∗^ *p* < 0.01 were considered statistically significant compared to the control.

Moderate inhibition was observed with the n‐hexane fraction at 400 mg/kg (26.4 ± 2.09; 40% inhibition) and the ME at 200 mg/kg (31.6 ± 2.14; 28.18% inhibition). The dichloromethane fraction showed the least activity, with only 18.63% inhibition at 400 mg/kg. These results highlight the potent peripheral analgesic effects of the ME and ethyl acetate fraction, indicating the presence of active phytoconstituents responsible for the observed activity.

#### 3.2.3. Anti‐Pyretic Activity

The ME of *Desmos chinensis* exhibited significant, dose‐dependent anti‐pyretic effects in Brewer’s yeast‐induced pyrexia model (Table 5). At 400 mg/kg, ME normalized rectal temperature by the 4th hour (96.10 ± 0.12°F; 100% reduction), comparable to the standard paracetamol (96.52 ± 0.30°F; 97.94% reduction). At 200 mg/kg, ME also showed progressive temperature reduction, reaching 96.90 ± 0.06°F (98.9%) at the 4th hour (*p* < 0.001). Both doses significantly reduced fever from the 2nd hour onward, indicating strong and sustained anti‐pyretic activity. The control group showed no significant decrease in temperature over time. These findings confirm the ME’s potent, dose‐dependent anti‐pyretic effect, which is comparable to that of the standard drug at higher doses.

**TABLE 5:** Effect of *Desmos chinensis* methanolic leaves extract on Brewer’s yeast‐induced pyrexia in mice.

Test samples and dose (mg/kg)	Rectal temperature °F (mean ± SEM)					
	Test samples administration					
	Before administration		After administration (% of pyrexia reduction)			
	Normal	After 24 h	1st h	2nd h	3rd h	4th h
Control‐10 mL/kg	95.73 ± 0.44	101.10 ± 0.90	101.87 ± 0.70	102.33 ± 0.50	102.37 ± 0.60	102 ± 0.71
Paracetamol‐150	96.50 ± 0.45	100.13 ± 0.20	97.73 ± 0.49^∗∗∗^ (66.12%)	97.03 ± 0.58^∗∗∗^ (85.40%)	96.60 ± 0.47^∗∗∗^ (97.25%)	96.52 ± 0.30^∗∗∗^ (97.94%)
ME‐200	96.87 ± 0.44	99.6 ± 0.29	99.00 ± 0.32^∗∗^ (21.98%)	97.97 ± 0.45^∗∗∗^ (59.71%)	97.20 ± 0.35^∗∗∗^ (87.91%)	96.90 ± 0.06^∗∗∗^ (98.9%)
ME‐400	96.17 ± 0.22	100.17 ± 0.18	99.03 ± 0.44^∗∗^ (28.5%)	98.07 ± 0.48^∗∗∗^ (52.5%)	96.87 ± 0.20^∗∗∗^ (82.5%)	96.10 ± 0.12^∗∗∗^ (100%)

*Note:* Each value represents the mean ± SEM (*n* = 5). One‐way ANOVA followed by Dunnett’s *t*‐test.

Abbreviation: ME, methanolic extract.

^∗∗∗^ *p* < 0.001 and ^∗∗^ *p* < 0.01 were considered statistically significant compared to the control.

#### 3.2.4. Anti‐Inflammatory Activity by the Protein Denaturation Method

**FIGURE 2:**
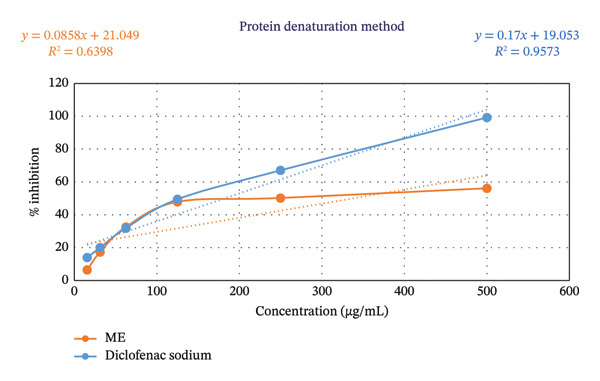
Evaluation of anti‐inflammatory activity of *D. chinensis* through the protein denaturation method.

The ME exhibited an apparent, dose‐dependent inhibition of protein denaturation (Table 6 and Figure 2). At the highest tested concentration (500 μg/mL), ME produced 56.18% inhibition, while at 250 and 125 μg/mL, it achieved 50.28% and 47.90% inhibition, respectively. Lower concentrations (62.5, 31.25, and 15.625 μg/mL) produced progressively lower activities of 32.60%, 17.34%, and 6.50%, respectively. In comparison, the reference drug, diclofenac sodium, showed markedly greater inhibition, reaching 99.24% at 500 μg/mL and 67.10% at 250 μg/mL. Nonlinear regression analysis yielded IC_50_ values of 337.42 μg/mL for ME and 182.05 μg/mL for diclofenac sodium, indicating that the standard drug was approximately 1.8‐fold more potent. These findings confirm that the ME exhibits significant, concentration‐dependent anti‐inflammatory activity, though with lower potency than diclofenac sodium.

**TABLE 6:** Absorbance and percent inhibition of methanolic extract (ME) of *D. chinensis* and diclofenac sodium.

Concentration (μg/mL)	ME		Diclofenac sodium	
	% inhibition	IC_50_	% inhibition	IC_50_
500	56.18	337.42	99.244	182.05
250	50.28		67.098	
125	47.90		49.463	
62.5	32.60		31.866	
31.25	17.34		20.049	
15.625	6.5		13.963	

#### 3.2.5. Antiarthritic Activity by HRBC Membrane Stabilization Method

**FIGURE 3:**
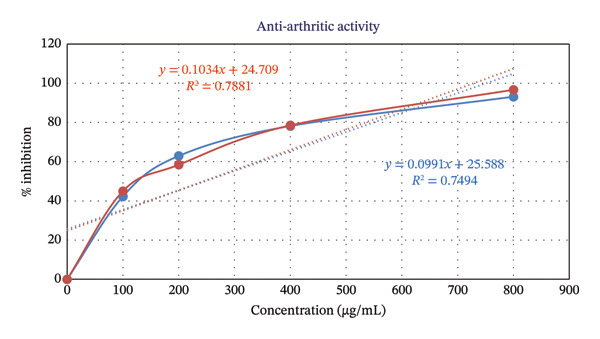
Evaluation of anti‐arthritic activity of the methanolic extract (ME) of *Desmos chinensis*.

The ME exhibited pronounced membrane stabilization activity in the HRBC assay (Table 7 and Figure 3). The percentage inhibition of hemolysis increased in a concentration‐dependent manner for both the sample and the standard, diclofenac sodium. At the highest tested concentration (800 μg/mL), the sample exhibited 93.16% inhibition, comparable to that of diclofenac sodium (96.69%). At lower concentrations, the sample also showed considerable protection of the HRBC membrane, with 78.21%, 62.93%, and 42.31% inhibition at 400, 200, and 100 μg/mL, respectively, compared to diclofenac sodium values of 78.53%, 58.44%, and 44.98% at the corresponding concentrations.

**TABLE 7:** Evaluation of anti‐arthritic activity of the methanolic extract (ME) of *D. chinensis* through HRBC membrane stabilization method.

Concentration (μg/mL)	Sample		Diclofenac sodium	
	% inhibition	IC_50_	% inhibition	IC_50_
800	93.16	246.34	96.688	244.593
400	78.21		78.526	
200	62.93		58.44	
100	42.31		44.979	
0	0		0	

The IC_50_ values further support this observation. The test sample demonstrated an IC_50_ of 246.34 μg/mL, which was close to that of diclofenac sodium (244.59 μg/mL), indicating that the sample possesses membrane‐stabilizing and anti‐arthritic potential comparable to that of the standard drug.

These findings highlight that the extract not only stabilized the erythrocyte membrane effectively but also produced a concentration‐dependent anti‐arthritic effect, suggesting that its mechanism of action may be similar to that of standard NSAIDs.

### 3.3. In Silico Study

#### 3.3.1. ADME and Drug‐Likeness Prediction

In silico ADME analysis was employed to assess the pharmacokinetic and toxicological profiles of 18 phytochemicals isolated from *D. chinensis* leaves (Table 8). Absorption (intestinal absorption and water solubility), distribution (volume of distribution and blood‐brain barrier penetration), metabolism (CYP3A4 substrate status), excretion (total clearance), and toxicity (AMES test for mutagenicity and hepatotoxicity) were the main areas of focus for the analysis. The overall potential of these compounds as orally active medication candidates was also determined by calculating expected oral bioavailability and evaluating drug‐likeness using Lipinski’s Rule of Five.

**TABLE 8:** ADME/T and drug‐likeliness properties of identified compounds of *D. chinensis*.

Compounds name	Absorption		Distribution		Metabolism	Excretion	Drug likeliness	Bioavailability
	Water solubility (log mol/L)	Intestinal absorption (human) (% absorbed)	VDss (human) (log L/kg)	BBB permeability (log BB)	CYP3A4 substrate	Total clearance (log mL/min/kg)		
β‐caryophyllene	−5.555	94.845	0.652	0.733	No	1.088	Yes	0.55
Phytol	−7.535	90.643	0.385	0.793	Yes	1.686	Yes	0.55
β‐sitosterol	−6.773	94.464	0.193	0.781	Yes	0.628	Yes	0.55
Stigmasterol	−6.682	94.97	0.178	0.771	Yes	0.618	Yes	0.55
Hexadecanoic acid	−5.562	92.004	−0.543	−0.111	Yes	1.763	Yes	0.85
Limonene	−3.568	95.898	0.396	0.732	No	0.213	Yes	0.55
α‐pinene	−3.733	96.041	0.667	0.791	No	0.043	Yes	0.55
Germacrene D	−5.682	95.59	0.544	0.723	No	1.42	Yes	0.55
1,8‐cineole (eucalyptol)	−2.63	96.505	0.491	0.368	No	1.009	Yes	0.55
Benzyl benzoate	−3.582	95.835	−0.046	0.282	Yes	0.727	Yes	0.55
β‐pinene	−4.191	95.525	0.685	0.818	No	0.03	Yes	0.55
Desmosflavone	−4.215	95.12	0.151	−0.131	Yes	0.456	Yes	0.55
Astilbin	−3.03	49.005	1.592	−1.199	No	−0.284	No	0.17
Quercitrin	−2.903	52.709	1.517	−1.495	No	0.364	No	0.17
Benzaldehyde	−1.198	96.246	0.027	0.082	No	0.243	Yes	0.55
Myrcene	−4.497	94.696	0.363	0.781	No	0.438	Yes	0.55
α‐humulene	0.438	94.682	0.505	0.663	No	1.282	Yes	0.55
Caryophyllene oxide	−4.433	95.421	0.586	0.654	No	0.905	Yes	0.55

#### 3.3.2. Toxicity Evaluation

Several indices, including immunotoxicity, toxicity class, LD_50_ (median lethal dose), hepatotoxicity, cytotoxicity, carcinogenicity, and mutagenicity, were evaluated to assess the toxicity of the docked phytocompounds. Table 9 summarizes the findings from this toxicity assessment.

**TABLE 9:** Toxicity profiles of selected compounds of *D. chinensis*.

Compound name	Predicted LD_50_ (mg/kg)	Predicted toxicity class	AMES toxicity	Acute oral toxicity	Hepatotoxicity	Carcinogenicity	Mutagenicity	Immunotoxicity	Cytotoxicity
β‐caryophyllene	5300	5	−	III	−	−	−	+	−
Phytol	5000	5	−	III	−	−	−	−	−
β‐sitosterol	890	4	−	I	−	−	−	+	−
Stigmasterol	890	4	−	I	−	−	−	+	−
Hexadecanoic acid	900	4	−	IV	−	−	−	−	−
Limonene	4400	5	−	III	−	−	−	−	−
α‐pinene	3700	5	−	III	−	−	−	−	−
Germacrene D	5300	5	−	III	−	−	−	+	−
1,8‐cineole (eucalyptol)	2480	5	−	III	−	−	−	−	−
Benzyl benzoate	1000	4	−	III	−	+	+	−	−
β‐pinene	4700	5	−	III	−	−	−	−	−
Desmosflavone	2570	5	−	III	−	−	−	−	−
Astilbin	2300	5	−	III	−	+	−	+	−
Quercitrin	5000	5	−	III	−	−	−	+	−
Benzaldehyde	28	2	−	III	−	+	−	−	−
Myrcene	5000	5	−	III	−	−	−	−	−
α‐humulene	3650	5	−	III	−	−	−	−	−
Caryophyllene oxide	5000	5	−	III	−	−	−	+	−

*Note:* Presence (+), absence (−).

#### 3.3.3. Molecular Docking Study

**FIGURE 4:**
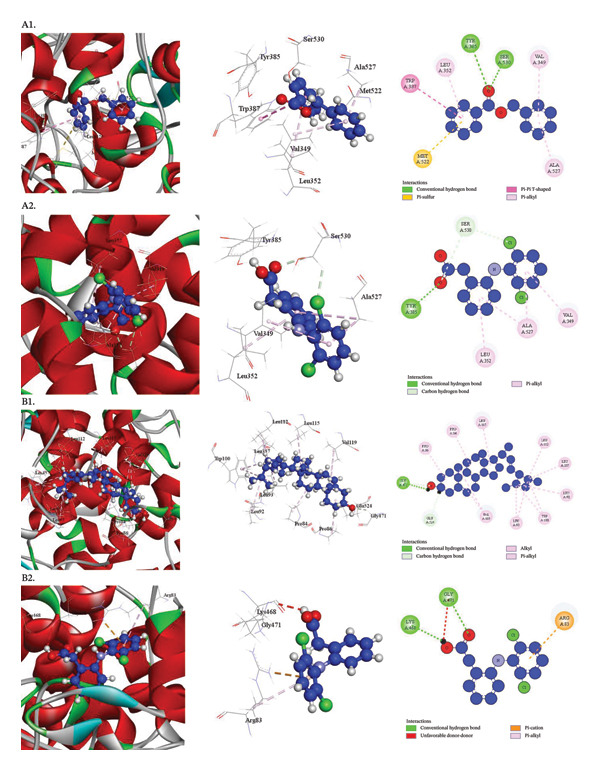
Docking interactions of phytochemicals against human cyclooxygenase‐2 (PDB ID: 5IKR): A1, Benzyl benzoate; A2, Diclofenac (standard) and human cyclooxygenase (hCOX)‐1 (PDB ID: 6Y3C): B1, Stigmasterol; B2, Diclofenac (standard).

**FIGURE 5:**
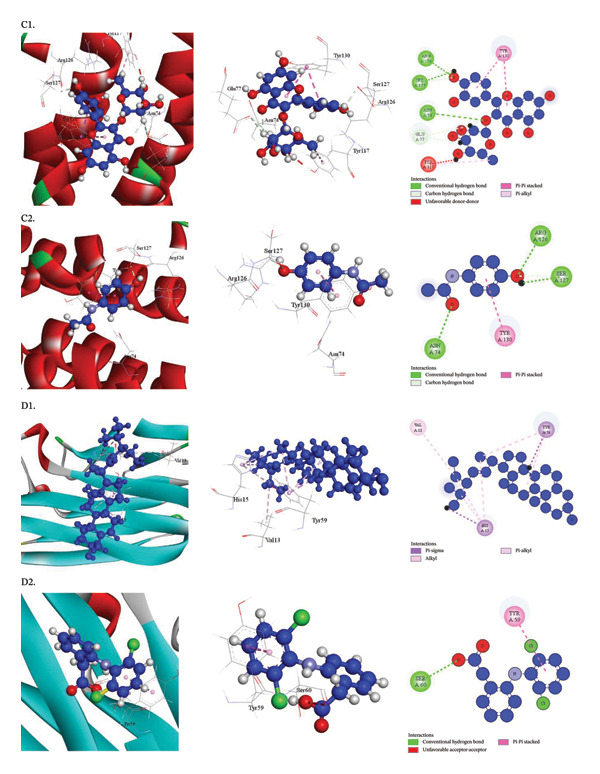
Docking interactions of phytochemicals against microsomal prostaglandin E synthase 1 (mPGES‐1) receptor (PDB ID: 4YK5): C1, Quercitrin; C2, Paracetamol (standard) and TNF‐alpha using a small molecule inhibitor (PDB ID: 2AZ5): D1, Stigmasterol; D2, Diclofenac (standard).

Human Cyclooxygenase‐2 (PDB ID: 5IKR), Human Cyclooxygenase (hCOX)‐1 (PDB ID: 6Y3C), Microsomal Prostaglandin E Synthesis 1 (mPGES‐1) (PDB ID: 4YK5), and TNF‐alpha with a small‐molecule inhibitor (PDB ID: 2AZ5) are among the receptors against which the docking scores of compounds from the *D. chinensis* are displayed in Table 10. Table 11 and Figures 4 and 5 provide additional information on how the best‐performing chemical from *D. chinensis* interacts with the amino acid residues of these proteins. The molecular docking results are further examined in these pictures, which also show how the bioactive chemicals bind to the target proteins’ active sites or other crucial regions, providing information on possible modes of action.

**TABLE 10:** Docking scores of the phytochemicals identified from *D. chinensis* for anti‐nociceptive, anti‐inflammatory, anti‐pyretic, and anti‐arthritic activity.

Compound name	PubChem ID	Binding affinity (kcal/mol)			
		Anti‐nociceptive (5IKR)	Anti‐inflammatory (6Y3C)	Anti‐pyretic (4YK5)	Anti‐arthritic (2AZ5)
β‐caryophyllene	5281515	−4.5	−6.1	−4.7	−5.5
Phytol	5280435	−6.5	−5.2	−3.8	−4.2
β‐sitosterol	222284	−5.3	−7.9	−5.4	−6.5
Stigmasterol	5280794	−5.3	**−8.5**	−5.7	**−7.1**
Hexadecanoic acid	985	−6.3	−4.5	−3.7	−3.8
Limonene	22311	−6.3	−6	−3.8	−4.5
α‐pinene	6654	−5.1	−4.6	−3.7	−4.2
Germacrene D	5317570	−7.7	−6.3	−4.5	−5.5
1,8‐cineole (eucalyptol)	2758	−4.3	−4.6	−3.9	−4.3
Benzyl benzoate	2345	**−7.8**	−5.5	−5.6	−5.6
β‐pinene	14896	−5.2	−4.6	−3.7	−4.2
Desmosflavone	369598	−7.5	−7.4	−5.7	−6.4
Astilbin	119258	−3.6	−8.1	−6	−6.9
Quercitrin	5280459	−4.7	−8.3	**−6.5**	−6.7
Benzaldehyde	240	−5.5	−5	−3.8	−3.7
Myrcene	31253	−5.8	−5.4	−3.9	−4.2
α‐humulene	5281520	−4.5	−5.9	−4.6	−5.4
Caryophyllene oxide	1742210	−5.7	−6.3	−4.8	−5.4
Standards (diclofenac, diclofenac, paracetamol, diclofenac)		−8.1	−6.2	−4.4	−5.7

*Note:* The bold value represents the best binding affinity for each target/protein.

**TABLE 11:** Binding affinity and in silico binding interactions of selected compounds of *D. chinensis* for anti‐nociceptive (PDB: 5ikr), anti‐inflammatory (PDB: 6y3c), anti‐pyretic (PDB: 4yk5), and anti‐arthritic (PDB: 2az5) activity.

Section	Protein ID	Compound	Binding affinity (kcal/mol)	Class of bonds	Amino acid residue
1	5ikr	Benzyl benzoate	−7.8	Conventional hydrogen bond	TYR385, SER530
				Pi‐sulfur	MET522
				Pi‐Pi T‐shaped	TRP387
				Pi‐alkyl	VAL349, ALA527, LEU352
		Diclofenac (standard)	−8.1	Conventional hydrogen bond	TYR385
				Carbon hydrogen bond	SER530 (2)
				Pi‐alkyl	LEU352, ALA527 (2), VAL349

2	6y3c	Stigmasterol	−8.5	Conventional hydrogen bond	GLY471
				Carbon hydrogen bond	GLY471, GLU524
				Alkyl	PRO84, PRO86, LEU115, VAL119 (2), LEU92, LEU93 (2), LEU112, LEU357, TRP100 (2)
		Diclofenac (standard)	−6.2	Conventional hydrogen bond	GLY471, LYS468
				Pi‐cation	ARG83
				Pi‐alkyl	ARG83

3	4yk5	Quercitrin	−6.5	Conventional hydrogen bond	ASN74, ARG126, SER127
				Carbon hydrogen bond	SER127, GLU77 (2)
				Pi‐Pi stacked	TYR130 (2)
				Pi‐alkyl	TYR117
		Paracetamol (standard)	−4.4	Conventional hydrogen bond	ASN74, ARG126, SER127
				Carbon hydrogen bond	SER127
				Pi‐Pi stacked	TYR130

4	2az5	Stigmasterol	−7.1	Pi‐sigma	TYR59, HIS15
				Alkyl	VAL13
				Pi‐alkyl	HIS15 (3), TYR59 (2)
		Diclofenac (standard)	−5.7	Conventional hydrogen bond	SER60
				Pi‐Pi stacked	TYR59

##### 3.3.3.1. Docking Analysis for Anti‐Nociceptive Activity

Regarding their anti‐nociceptive properties, each molecule showed affinity for Human Cyclooxygenase‐2 (PDB ID: 5IKR). Interestingly, the maximum binding affinity was −7.8 kcal/mol for benzoyl benzoate, which was nearly identical to the reference medication diclofenac (−8.1 kcal/mol) [Table 11 (Section 1), Figure 4]. Benzyl benzoate formed seven interactions with key amino acid residues of the receptor, including TYR385, SER530, MET522, TRP387, VAL349, ALA527, and LEU352, at modest intermolecular distances, as determined by a thorough examination of the docking process. The chemical may be a good option for further research into its anti‐nociceptive properties due to this significant interaction, which suggests a high binding affinity for the Cyclooxygenase‐2 receptor.

##### 3.3.3.2. Docking Analysis for Anti‐Inflammatory Activity

By demonstrating affinity for the human cyclooxygenase (hCOX)‐1 (PDB ID: 6Y3C), each compound showed anti‐inflammatory action. The reference drug, diclofenac, had a binding affinity of −6.2 kcal/mol, whereas stigmasterol had a higher binding affinity of −8.5 kcal/mol [Table 11 (Section 2), Figure 4]. A thorough examination of the docking process showed that, at short intermolecular distances, stigmasterol forged 15 connections with the receptor’s amino acid residues. These interactions demonstrate the compound’s remarkable binding affinity for the human cyclooxygenase (hCOX)‐1 active site, underscoring its promise as a powerful anti‐inflammatory drug.

##### 3.3.3.3. Docking Analysis for Anti‐Pyretic Activity

The ability of each item to interact with the Microsomal prostaglandin E synthase 1 (mPGES‐1) receptor (PDB ID: 4YK5) was assessed. With a binding affinity of −6.5 kcal/mol, quercitrin outperformed the reference drug, paracetamol (−4.4 kcal/mol), among the compounds that were chosen. Quercitrin demonstrated a substantial binding affinity to the active site of the Microsomal prostaglandin E synthase 1 enzyme by forming nine contacts with ASN74, ARG126, SER127, SER127, GLU77 (2), TYR130 (2), and TYR117 at modest intermolecular distances, according to a rigorous study of the docking process. Table 11 (Section 3) and Figure 5 indicate that the chemical has significant promise for efficiently targeting the Microsomal prostaglandin E synthase 1 (mPGES‐1) enzyme.

##### 3.3.3.4. Docking Analysis for Anti‐Arthritic Activity

Anti‐arthritic action was shown by each chemical that was able to interact with the TNF‐alpha using a small‐molecule inhibitor (PDB ID: 2AZ5). The binding affinity of stigmasterol was notably higher at −7.1 kcal/mol than that of the reference medication, diclofenac (−5.7 kcal/mol). The best‐performing compounds demonstrated a considerable affinity for the TNF‐alpha active site by generating significant interactions with amino acid residues, such as TYR59, HIS15, VAL13, HIS15 (3), and TYR59 (2), at short intermolecular distances. According to Table 11 (Section 4) and Figure 5, the chemical shows promise as an anti‐arthritic drug.

### 3.4. PASS Prediction

PASS prediction analysis was employed to assess the potential antinociceptive, anti‐inflammatory, anti‐pyretic, and anti‐arthritic activities of 18 selected compounds from *D. chinensis*. The results of this analysis are shown in Table 12.

**TABLE 12:** PASS prediction analysis of pharmacologically active compound of *D. chinensis*.

Compound name	Biological activity							
	Anti‐nociceptive		Anti‐inflammatory		Anti‐pyretic		Anti‐arthritic	
	Pa	Pi	Pa	Pi	Pa	Pi	Pa	Pi
β‐caryophyllene	0.414	0.099	0.745	0.011	—	—	0.482	0.058
Phytol	0.300	0.182	0.458	0.070	—	—	0.387	0.091
β‐sitosterol	0.558	0.014	0.467	0.067	—	—	0.463	0.064
Stigmasterol	**0.601**	0.008	0.542	0.045	—	—	0.490	0.056
Hexadecanoic acid	0.526	0.023	0.515	0.052	**0.497**	0.013	0.528	0.046
Limonene	0.452	0.069	0.610	0.029	—	—	0.420	0.078
α‐pinene	0.495	0.039	0.490	0.060	—	—	0.634	0.026
Germacrene D	0.392	0.116	0.457	0.070	—	—	0.318	0.126
1,8‐cineole (eucalyptol)	0.446	0.073	0.327	0.046	—	—	0.647	0.024
Benzyl benzoate	0.524	0.024	0.459	0.070	0.295	0.047	0.615	0.029
β‐pinene	0.448	0.072	0.611	0.029	—	—	0.591	0.033
Desmosflavone	0.264	0.212	0.690	0.017	0.242	0.078	0.702	0.017
Astilbin	0.517	0.027	0.628	0.026	0.210	0.104	0.372	0.098
Quercitrin	0.461	0.062	0.754	0.010	0.256	0.067	0.474	0.060
Benzaldehyde	0.482	0.048	0.415	0.019	0.307	0.043	**0.732**	0.014
Myrcene	0.425	0.090	0.288	0.172	—	—	0.502	0.053
α‐humulene	0.342	0.151	0.741	0.011	—	—	0.541	0.043
Caryophyllene oxide	0.424	0.091	**0.759**	0.009	—	—	0.340	0.114

*Note:* The bold values represent that Pa > Pi (a higher likelihood of biological activity where Pa ≥ 0.5).

## 4. Discussion

Natural products have garnered growing global interest due to their rich diversity of bioactive constituents and relatively low toxicity [46]. Therefore, the present study was undertaken to evaluate the potential analgesic, anti‐pyretic, and anti‐inflammatory activities of the ME of *Desmos chinensis* and its derived fractions, using a comprehensive approach that integrates in vivo, in vitro, and in silico methodologies.

The acetic acid–induced writhing test to evaluate peripheral analgesic effect in mice is a well‐established visceral pain model for screening both opioid and non‐opioid analgesics. This method involves intraperitoneal injection of acetic acid, which causes chemical irritation of the peritoneum and triggers a characteristic nociceptive response marked by abdominal constrictions (writhing) [47]. The pain response is mediated by the release of endogenous mediators such as bradykinin, prostaglandins, and other pro‐inflammatory substances that sensitize peripheral nociceptors [48]. In this study, the crude ME and the ethyl acetate fraction of *Desmos chinensis* administered at 400 mg/kg produced a significant reduction in the number of writhes, indicating potent peripheral analgesic activity. The reference drug diclofenac sodium also showed marked protection against acetic acid–induced pain, confirming the validity of the model. These findings suggest that the extract and its fraction exert their analgesic effects, at least in part, by inhibiting inflammatory mediators involved in peripheral nociception. Phytochemical screening of the ME of *D. chinensis* revealed the presence of flavonoids and alkaloids, both of which have been widely associated with analgesic and anti‐inflammatory activities in various experimental models. Numerous studies have demonstrated that flavonoids and other polyphenolic compounds modulate pain and inflammation through multiple pathways, including the inhibition of pro‐inflammatory mediators such as prostaglandins [49, 50]. Flavonoids, key in prostaglandin synthesis, are known to interfere with cyclooxygenase (COX) enzyme activity and may also influence opioidergic signaling pathways involved in pain modulation. These findings suggest that the observed peripheral analgesic effect of *D. chinensis* may be attributed to its flavonoid content, potentially acting through the inhibition of peritoneal nociceptive receptors and COX‐mediated inflammatory pathways [51].

Subcutaneous injection of Brewer’s yeast induces pyrexia by stimulating the synthesis of prostaglandins, particularly PGE_2_, in the hypothalamus, making it a well‐established model for evaluating anti‐pyretic agents [52]. This type of fever, known as pathogenic or endogenous pyrexia, is mediated by inflammatory cytokines and prostaglandins, which elevate the thermoregulatory set point [53]. The anti‐pyretic effect of many drugs, such as paracetamol, is attributed to the inhibition of cyclooxygenase (COX) enzymes, thereby suppressing prostaglandin production [54]. In this study, intraperitoneal administration of the ME of *D. chinensis* significantly reduced rectal temperature in yeast‐induced febrile mice, indicating potent anti‐pyretic activity. This effect may be mediated through the inhibition of prostaglandin synthesis via COX enzyme blockade. Phytochemical screening revealed the presence of flavonoids and alkaloids in the extract, both of which are known to contribute to anti‐pyretic effects. Flavonoids have been shown to inhibit COX activity and reduce prostaglandin levels, while alkaloids from plants such as *Hunteria zeylanica* have also demonstrated anti‐pyretic properties [51]. Therefore, the observed anti‐pyretic effect of *D. chinensis* is likely due to the synergistic action of these bioactive constituents, particularly flavonoids and alkaloids, which interfere with key mediators of the febrile response.

The ME of *D. chinensis* was evaluated for its ability to inhibit protein denaturation in an egg albumin assay. The results demonstrated significant concentration‐dependent anti‐inflammatory activity, albeit with lower potency than the standard drug diclofenac sodium. Protein denaturation is a well‐established model of inflammation, particularly relevant to conditions such as rheumatoid arthritis, where tissue damage and autoantigen formation occur due to structural changes in endogenous proteins [55, 56]. Inhibition of protein denaturation has been proposed as an early mechanism of action for NSAIDs, suggested by Mizushima and Kobayashi [56] before the discovery of their cyclooxygenase (COX) inhibitory effects by Vane [57]. This protective effect may contribute to antirheumatic activity by preventing the initiation of the inflammatory cascade. Consistent with these findings, several plant extracts have shown dose‐dependent inhibition of protein denaturation: Gambhire et al. [58] reported anti‐inflammatory effects of *Murraya koenigii* methanol extract via membrane stabilization and protein protection; Umapathy [59] demonstrated similar effects with *Albuca setosa* aqueous extract, including reduced WBC migration, and Chopade et al. [60] linked the anti‐inflammatory activity of *Phyllanthus amarus* to its ability to prevent thermal protein denaturation. Collectively, these findings suggest that ME‐DC exerts its in vitro anti‐inflammatory effect, at least in part, by inhibiting protein denaturation. This supports the potential therapeutic use of *D. chinensis* as a natural anti‐inflammatory agent, warranting further investigation into its active constituents and mechanisms.

The in vitro anti‐arthritic activity of *Desmos chinensis* extracts was evaluated using the HRBC membrane stabilization assay. The ME demonstrated significantly stronger membrane‐stabilizing effects compared to the standard drug diclofenac sodium, indicating potent anti‐arthritic potential. This assay is based on the principle that NSAIDs protect erythrocyte membranes from hypotonic stress–induced lysis by stabilizing the lipid bilayer, mirroring their ability to stabilize lysosomal membranes involved in inflammatory processes [61]. Lysosomal membrane rupture during inflammation releases hydrolytic enzymes that damage surrounding tissues; thus, agents that prevent membrane destabilization can mitigate inflammatory symptoms. Anti‐inflammatory compounds are known to bind to erythrocyte membranes, altering surface charge and enhancing structural integrity, thereby reducing hemolysis under osmotic stress [62]. Several herbal extracts and flavonoids have been reported to exert strong membrane‐stabilizing effects in vitro and in vivo, supporting their anti‐inflammatory and anti‐arthritic actions [63]. Phytochemical analysis confirmed the presence of flavonoids in the ME of *D. chinensis*, which likely contribute to its membrane‐stabilizing and anti‐arthritic activity. Flavonoids are known to interact with membrane lipids and proteins, preventing oxidative and thermal damage [64, 65]. These findings suggest that the extract’s potent HRBC membrane‐stabilizing effects may translate into significant anti‐inflammatory and disease‐modifying effects in conditions such as rheumatoid arthritis.

Molecular docking is a widely used computational approach for predicting ligand‐target interactions and gaining deeper insights into the biological activities of natural compounds. It also helps elucidate potential binding mechanisms within protein active sites. In this study, molecular docking was employed to validate and support the observed biological activities, enhancing our understanding of their underlying molecular basis [17]. Eighteen selected phytochemicals from *D. chinensis* were chosen for a comprehensive docking analysis. These compounds were docked against four key protein targets associated with pain, inflammation, fever, and arthritis: Human Cyclooxygenase‐2 (PDB ID: 5IKR), Human Cyclooxygenase‐1 (hCOX‐1, PDB ID: 6Y3C), Microsomal Prostaglandin E Synthase‐1 (mPGES‐1, PDB ID: 4YK5), and TNF‐alpha (PDB ID: 2AZ5) in complex with a small‐molecule inhibitor. The docking scores indicated that the formation of non‐covalent interactions, particularly hydrophobic contacts and hydrogen bonds with critical amino acid residues, plays a vital role in stabilizing ligands within the binding pockets. The results revealed that multiple amino acid residues actively contribute to these interactions, facilitating strong ligand–protein binding. Among the tested compounds, benzyl benzoate, Stigmasterol, and Quercitrin exhibited the highest binding affinities across all four target proteins, suggesting their potential as multi‐target agents with promising anti‐nociceptive, anti‐inflammatory, anti‐pyretic, and anti‐arthritic properties.

The pharmacokinetic and toxicological parameters of the compounds have been examined in relation to molecular docking results against Human Cyclooxygenase‐2, Human Cyclooxygenase (hCOX)‐1, Microsomal Prostaglandin E Synthase 1 (mPGES‐1), and TNF‐alpha, using a small‐molecule inhibitor. Every chemical complied with Lipinski’s guidelines for drug likelihood. As a result, the establishment of a novel pharmacological agent greatly benefits from these interpretations of detected compounds. The results suggest that the compounds are safe for oral use and comply with Lipinski’s guidelines, which indicate that they may function as promising therapeutic candidates [29]. Additionally, as medication safety is a crucial component of a quality medicinal product, we assessed the toxicological parameters of the assigned plant compounds using the admetSAR, pKCSM, and protox online tool [66, 67].

The PASS (Prediction of Activity Spectra for Substances) is a computational method that estimates the biological activities of chemical compounds by simulating their behavior across various bioactivity levels. In this study, a compound is considered likely to exhibit a specific biological effect if its Pa (probability “to be active”) value exceeds its Pi (probability “to be inactive”) value [68]. This analysis provided valuable insights into the potential pharmacological properties of the selected phytochemicals. The observed results may be attributed to the combined contribution of multiple phytochemicals, both previously reported and newly identified, suggesting a synergistic or additive effect that enhances the overall biological activity. Stigmasterol, caryophyllene oxide, hexadecanoic acid, and benzaldehyde emerged as the top‐scoring compounds in PASS prediction analysis for antinociceptive, anti‐inflammatory, anti‐pyretic, and anti‐arthritic activities, respectively (Table 12). These findings were well aligned with the outcomes of the corresponding in vivo and in vitro pharmacological tests, reinforcing the biological relevance of these phytoconstituents and supporting their potential contribution to the observed therapeutic effects of the extract.

## 5. Conclusion

The present study provides the first integrated in vivo, in vitro, and in silico evidence supporting the analgesic, anti‐pyretic, anti‐inflammatory, and anti‐arthritic potential of the methanolic leaf extract of *Desmos chinensis*. The extract and its solvent fractions significantly reduced acetic acid–induced writhing, suppressed Brewer’s yeast–induced pyrexia, and inhibited protein denaturation and HRBC membrane destabilization in a dose‐dependent manner. These pharmacological effects were further corroborated by molecular docking analyses, which revealed favorable binding affinities of major phytochemicals toward COX‐1, COX‐2, mPGES‐1, and TNF‐α, suggesting plausible mechanistic targets underlying the observed bioactivities.

Despite these promising findings, several limitations should be acknowledged. The study did not include isolation and quantification of individual active compounds, nor did it include in vivo anti‐arthritic models or long‐term toxicity evaluations. In addition, mechanistic validation at the molecular and cellular levels (e.g., cytokine profiling, COX‐2 expression, and signaling pathway analysis) remains unexplored.

Future research should focus on bioassay‐guided fractionation, structural characterization of lead compounds, pharmacokinetic and chronic toxicity studies, and validation using disease‐specific inflammatory and arthritic animal models. Furthermore, integrating transcriptomic or proteomic approaches would help clarify the molecular mechanisms of action and support the development of *D. chinensis* as a potential phytotherapeutic or nutraceutical candidate for inflammation‐related disorders.

## Author Contributions

Nigar Sultana Prithy: conceptualization (equal); data curation (equal); formal analysis (equal); investigation (equal); methodology (equal); software (equal); writing–original draft (lead); writing–review and editing (equal). Ainun Nahar: conceptualization (equal); data curation (equal); formal analysis (equal); investigation (equal); methodology (equal); software (equal); writing–original draft (supporting); writing–review and editing (equal). Md. Jahirul Islam Mamun: data curation (equal); formal analysis (equal); software (equal); writing–original draft (supporting); writing–review and editing (equal). Md. Hossain Rasel: data curation (equal); formal analysis (supporting); writing–original draft (supporting); writing–review and editing (supporting). Fahmida Akter: data curation (equal); formal analysis (equal); writing–review and editing (supporting). Kazi Sanjida Tahrim: formal analysis (supporting); writing–original draft (supporting); writing–review and editing (supporting). Raina Islam: writing original draft (supporting); writing–review and editing (supporting). Md. Emdadul Hossain Emon: writing original draft (supporting); writing–review and editing (supporting). Abu Bin Ihsan: writing–original draft (supporting); writing–review and editing (equal); supervision (equal).

## Funding

No funding was received for this manuscript.

## Ethics Statement

All experimental procedures involving humans andanimals were conducted following internationally accepted ethical standards.The collection of human blood samples was approved by the Institutional EthicsCommittee of Eastern University (Approval No: IERB‐2024‐0005) and performed inaccordance with the principles of the Declarationof Helsinki. Written informed consent was obtained from all volunteersprior to blood collection, and participants had not taken any non‐steroidalanti‐inflammatory drugs (NSAIDs) for at least two weeks before the study.Animal experiments were approved by the Institutional EthicsReview Board (IERB) of EasternUniversity (Approval No: IERB‐2024‐0005) and conducted according to the ARRIVE Guidelines 2.0 and the Guide for the Care and Use of LaboratoryAnimals. At the end of the experimental procedures, animals werehumanely euthanized by intraperitoneal administration of an overdose ofketamine (150 mg/kg) and xylazine (15 mg/kg), following the AVMA Guidelines for the Euthanasia of Animalsto minimize animal suffering.

## Conflicts of Interest

The authors declare no conflicts of interest.

## Data Availability

Data are available upon request from the authors.
